# TNF-α Mediated Increase of HIF-1α Inhibits VASP Expression, Which Reduces Alveolar-Capillary Barrier Function during Acute Lung Injury (ALI)

**DOI:** 10.1371/journal.pone.0102967

**Published:** 2014-07-22

**Authors:** Mengjie Tang, Yihao Tian, Doulin Li, Jiawei Lv, Qun Li, Changchun Kuang, Pengchao Hu, Ying Wang, Jing Wang, Ke Su, Lei Wei

**Affiliations:** 1 Department of Pathology and Pathophysiology, Research Center of Food and Drug Evaluation, School of Basic Medical Sciences, Wuhan University, Wuhan, Hubei, PR China; 2 Hunan Provincial Tumor Hospital, the Affiliated Tumor Hospital of Xiangya Medical School of Central South University, Changsha, Hunan, PR China; 3 Zhongnan Hospital of Wuhan University, the Second College of Clinical Medicine of Wuhan University, Wuhan, Hubei, PR China; 4 Renmin Hospital of Wuhan University, the First College of Clinical Medicine of Wuhan University, Wuhan, Hubei, PR China; 5 Renmin Hospital of Wuhan University, Division of Nephrology, Wuhan, Hubei, PR China; Emory University School of Medicine, United States of America

## Abstract

Acute lung injury (ALI) is an inflammatory disorder associated with reduced alveolar-capillary barrier function and increased pulmonary vascular permeability. Vasodilator-stimulated phosphoprotein (VASP) is widely associated with all types of modulations of cytoskeleton rearrangement-dependent cellular morphology and function, such as adhesion, shrinkage, and permeability. The present studies were conducted to investigate the effects and mechanisms by which tumor necrosis factor-alpha (TNF-α) increases the tight junction permeability in lung tissue associated with acute lung inflammation. After incubating A549 cells for 24 hours with different concentrations (0–100 ng/mL) of TNF-α, 0.1 to 8 ng/mL TNF-α exhibited no significant effect on cell viability compared with the 0 ng/mL TNF-α group (control group). However, 10 ng/mL and 100 ng/mL TNF-α dramatically inhibited the viability of A549 cells compared with the control group (*p<0.05). Monolayer cell permeability assay results indicated that A549 cells incubated with 10 ng/mL TNF-α for 24 hours displayed significantly increased cell permeability (*p<0.05). Moreover, the inhibition of VASP expression increased the cell permeability (*p<0.05). Pretreating A549 cells with cobalt chloride (to mimic a hypoxia environment) increased protein expression level of hypoxia inducible factor-1α (HIF-1α) (*p<0.05), whereas protein expression level of VASP decreased significantly (*p<0.05). In LPS-induced ALI mice, the concentrations of TNF-α in lung tissues and serum significantly increased at one hour, and the value reached a peak at four hours. Moreover, the Evans Blue absorption value of the mouse lung tissues reached a peak at four hours. The HIF-1α protein expression level in mouse lung tissues increased significantly at four hours and eight hours (**p<0.001), whereas the VASP protein expression level decreased significantly (**p<0.01). Taken together, our data demonstrate that HIF-1α acts downstream of TNF-α to inhibit VASP expression and to modulate the acute pulmonary inflammation process, and these molecules play an important role in the impairment of the alveolar-capillary barrier.

## Introduction

Acute lung injury (ALI) is characterized by pneumonedema and pulmonary closure caused by diffuse alveolar-capillary membrane damage, which appears after the body suffers injuries such as severe infection, trauma, or shock [1]. The normal alveolar respiratory membrane is divided into six layers, which can be successively identified from the alveolar inner surface to the outer surface under an electron microscope: a liquid layer with alveolar surfactant, the alveolar epithelium, the epithelial basement membrane, the matrix layer between the alveoli and capillary, the capillary basement membrane and the capillary endothelial layer. The permeability of the alveolar epithelium and capillary endothelium is the most crucial aspect of the six layers during the process of ALI, especially the permeability of the alveolar epithelium, an increase of which will finally lead to acute respiratory distress syndrome (ARDS) [2], [3]. Although the underlying mechanism has not yet been fully elucidated, inflammation has recently been reported as a crucial factor in causing ALI [4].

In the inflammation environment, the permeability of the pulmonary vascular membrane increases because of the excessive local pulmonary production of inflammatory factors, such as tumor necrosis factor-alpha (TNF-α), interleukin-6 (IL-6) and interleukin-1β (IL-1β). Finally, fatal pneumonedema occurs, which is characterized by refractory hypoxemia, respiratory distress and respiratory failure, which results in a serious threat to life [5]. TNF-α is a major proinflammatory and immunomodulatory factor in the human body that participates in both acute and chronic inflammation processes. TNF-α secretion increases and can be detected in lung tissues both in ALI patients and mice. TNF-α promotes the expression of adhesion molecules in vascular endothelial cells, stimulates the activation and migration of vascular endothelial cells, fibroblasts and monocytes/macrophages and initiates the inflammatory reaction by inducing the secretion of cytokines [6], [7].

Recent studies have demonstrated that the transcription of hypoxia inducible factor-1 alpha (HIF-1α) can be activated by inflammatory cytokines, such as TNF-α, IL-1β, prostaglandin E_2_ (PGE_2_) and lipopolysaccharide (LPS), which suggests that HIF is closely implicated in the inflammatory process [8], [9]. HIF has a wide target spectrum of genes that are associated with tumor growth, proliferation, the metastasis and inflammation process, inflammatory cell metabolism, chemotropism, survival, etc. [10], [11]. In addition, HIF is a major factor mediating the mammalian hypoxia reaction. It is a heterodimer that consists of hypoxic response factor HIF-1α and the constitutively expressed HIF-1β. HIF-1α can bind to the transcription initiation complex to influence the transcriptional expression of genes [12], [13].

Vasodilator-stimulated phosphoprotein (VASP) expression is widely associated with various modulations of cellular morphology and functions via the rearrangement of the cytoskeleton, such as polarity, conglutination, shrinkage, movement and even cell-cell junctions. For example, Peter Rosenberger's study indicated that inhibiting VASP expression in HMEC-1 during hypoxia could induce pneumonic edema in mice. Moreover, Ke Su's study identified that a decrease of VASP mRNA and protein expression mediated the TNF-α-induced adhesion and proliferation inhibition of breast cancer MCF-7 cells [14], [15]. TNF-α and IL-1β alter vascular permeability by regulating tight junctions and gap junctions, and they repress the expression of Connexin (Cx) in gap junctions at the transcriptional level by activating nuclear factor-κB (NF-κB) or HIF-1α [16]–[19]. Furthermore, recent studies have demonstrated that HIF-1α dramatically represses VASP transcription by directly binding to the VASP promoter [15]. However, the mechanism by which the inflammatory environment affects tight junctions in ALI still remains unexplored.

In this study, we illustrate how the TNF-α/HIF-1α/VASP pathway functions in tight junctions to regulate the permeability of the alveolar-capillary membrane and to cause ALI at both the cellular and organismal levels.

## Materials and Methods

### Cell Culture

Human lung adenocarcinoma A549 cells were purchased from the China Center for Type Culture Collection, which originated from type II human alveolar epithelial cells. A549 cells were cultured in RPMI-1640 medium (HyClone, USA) containing 10% fetal bovine serum (FBS; HyClone, USA), 50 U/mL penicillin-G and 50 µg of streptomycin at 37°C in a humidified incubator that was supplemented with 5% CO_2_. The cells were then passaged at 80–90% confluence and digested with 0.25% trypsin.

### MTT Assay

The trypsinized cells were seeded in 96-well plates (1×10^3^∼1×10^4^ cells/well) and grown overnight in complete RPMI-1640 medium. The next day, the culture was washed, and A549 cells in experimental groups were treated with different concentrations (0 – 100 ng/mL) of TNF-α (Invitrogen, USA). The same volume of nutrient medium was added into the control group. After 24 hours, the number of viable cells was determined by the addition of 3-(4,5-dimethylthiazol-2-yl)-2,5-diphenyltetrazoliumbromide (MTT; Sigma, USA). The absorption value of each well was measured with an enzyme-linked immuno-assay. Three independent experiments were performed with triplicate wells.

### RNA Interference

The resuspended cells were cultured in 6-well culture plates for 24 hours, and the cells were then re-cultured in antibiotic-free and serum-free RPMI-1640 medium for at least 8 hours before TNF-α incubation. The cells were transiently transfected with Turbofect Transfection Reagent (Roche, SC). The shRNA duplexes were designed against VASP (GenBank accession no. BC038224) with the following sequences: 5′-TGCTGTAAAGCATCACAGTGGCCCGGGTTTTGGCCACTGACTGACCCGGGCCAGTGATGCTTTA -3′ and were inserted into the pcDNA6.2-GW/EmGFP vector (Invitrogen, USA) to make pcDNA6.2-GW/EmGFP-miR-VASP. The siRNA duplex oligonucleotides to HIF-1α (GenBank accession no. NM_001530) were synthesized by Shanghai GenePharma (Shanghai, China). The sequence for HIF-1a-siRNA was as follows: 5′-CUGAUGACCAGCAACUUGAdTdT -3′. A scrambled-siRNA (5′- AGUUCAACGACCAGUAGUCdTdT-3′) was used in all experiments. Scrambled shRNA was obtained from Invitrogen and used as a negative control in all experiments. After 8–9 hours, the transfection reagent was removed. Medium with antibiotic and serum was added to the plates, and the cells were sequentially cultured for 24 hours.

### Monolayer Cell Paracellular Permeability Assay

A monolayer cell permeability assay was performed with the Transwell system, which allowed FITC-Dextran to filter through a 0.4 µm pore size polycarbonate membrane that supported a monolayer of cells. Ten microliters of gelatin were added to the upper transwell chamber (Costar, the diameter was 6.5 nm, and the pore size was 0.4 µm, USA), and the mixture was incubated overnight. Each upper chamber was hydrated with 200 µL of nutrient medium for 15–20 minutes. The resuspended cells (1×10^5^ cells/well, 200 µL) were plated in the upper chambers of a Transwell, and 600 µL of RPMI-1640 medium that was supplemented with 10% fetal bovine serum was added into the lower chamber in a humidified atmosphere for 24 hours. Subsequently, 10 ng/mL TNF-α was added to the upper chamber. After 24 hours, 200 µL of 100 ng/mL FITC-Dextran (Sigma, USA) was added to the upper chamber for 40 minutes. Nutrient medium (100 µL) from both the upper chamber and the lower chamber was removed and added to black 96-well plates. The absorption value was detected by a fluorescence microplate reader with an excitation wavelength of 485 nm and absorption wavelength of 535 nm. The cell permeability was measured using the coefficient Pa, which was calculated with the following formula: Pa = [L]/t ×1/A×v/[A], where [L] stands for the concentration of FITC-dextran in the lower chamber (marked with fluorescence); t stands for time, measured in seconds; A stands for the area of filter membrane, measured in cm; v stands for the volume of liquid in the bottom chamber; [A] stands for the concentration of FITC-Dextran in the lower chamber. All tests were repeated three times and statistically analyzed.

### Western Blotting

Cells or mouse lung tissues were lysed in modified RIPA buffer. The protein concentration was measured with the BCA protein assay kit (Pierce, USA). Equal amounts of total protein (20 µg) were loaded and run on a 5% (v/v) SDS-polyacrylamide stacking gel and 10% (v/v) SDS-polyacrylamide gradient gel and then transferred to PVDF membranes (Roche, SC). Membranes were blocked for 1 hour at room temperature with 5% powdered skim milk in Tris-buffered saline (TBS) with 0.05% Tween 20 (TBST) and then probed with VASP antibody (1∶800 dilution, Alexis, San Diego, CA, USA), HIF-1α antibody (1∶1000 dilution, Abcam, Cambridge, UK) or GAPDH antibody (1∶200 dilution, SC) at 4°C overnight. After incubation with horseradish peroxidase (HRP)-linked secondary antibodies (1∶5000, Santa Cruz Biotechnology, Santa Cruz, CA, USA) for 2 hours at room temperature, the blots were detected with AP. The experiments were repeated three times.

### Reverse-transcription Polymerase Chain Reaction (RT-PCR)

Total RNA was extracted from cells with Trizol (Invitrogen). Total RNA (2 mg) was used for first-strand cDNA synthesis with a RevertAid First Strand cDNA Synthesis Kit (Fermentas, Vilnius, LTU). Quantitative PCR was performed in the presence of SYBR green using a 7500 Fast Real-Time PCR System. All PCR reactions were run in triplicate and repeated at least three times. Differences were calculated according to the ^△△^Ct relative quantization method using the β-actin gene for calibration. The primers for human VASP were 5′- AAAGTCAGCAAGCAGGAGGA-3′ and 5′- ATTCATCCTTGGGGGTTTTC-3′. The primers for human HIF-1α were 5′ - GAAAGCGCAAGTCCTCAAAG -3′ and 5′-TGGGTAGGAGATGGAGATGC-3′. The primers for β-actin were 5′- CATTAAGGAGAAGCTGTGCT-3′ and 5′-GTTGAAGGTAGTTTCGTGGA-3′.

### Animal Model

This study was performed with the approval of the Wuhan University Medical Ethical Committee.

Ten Balb/c mice (5–6 weeks, weight 22–25 g, purchased from Wuhan University Center for Animal Experiment, Permit Number: SCXK 2008–0004) were randomly divided into two groups with five mice per group. The mice in each experimental group were peritoneally injected with 0.01 mg/g LPS (O111:B4, Sigma, USA) fluid per mouse to model ALI, and the mice in the control group were injected with the same dose of saline. After 0–8 hours, all left lung tissues were carefully separated and then fixed in 4% paraformaldehyde for HE staining or homogenized in PBS for the enzyme-linked immunosorbent assay (ELISA). All right lung tissues were separated to measure the wet-to-dry weight ratio as follows. The moisture of the tissues was wicked away with filter paper to immediately measure the wet weight with an electronic balance. The lung tissues were then placed in a 60°C thermotank for 24 hours until reaching a constant weight, after which the dry weight was measured. Finally, the wet/dry weight ratio (W/D) was calculated with the following formula: (W/D)  =  (wet weight/dry weight) × 100% [20], [21]. The experiments were repeated three times.

### ELISA Assay

Lung lobes (0.1 mg) were homogenized in 1 mL of PBS using a tissue homogenizer. The homogenate was centrifuged at 3000 rpm for 5 minutes. The supernatant was then harvested and preserved at −20°C for the ELISA assay.

The supernatant and mouse serum samples were subjected to ELISA analysis to measure the TNF-α secretion level. All operations were conducted strictly according to the Ebioscience protocol.

### Lung Barrier Permeability Assay [22]

The Balb/c mice in the experimental group were peritoneally injected with 0.01 mg/g LPS fluid per mouse to model ALI for 0, 2, 4 and 8 hours, whereas the mice in the control group were injected with the same dose of saline. The lung barrier function of the mice was assayed with the Evans blue (EB, Sigma, USA) dye extra-barrier technique to determine the alveolar epithelium integrity. Thirty minutes before being sacrificed, EB was injected into the mice via the tail vein at a dose of 50 mg/kg. The mice were then sacrificed, and the lungs were removed to prepare the homogenate. Formamide was added to the homogenate. The homogenate was then incubated at 37°C for 24 hours and centrifuged to obtain the supernatant. Subsequently, the absorption of EB in the supernatant was detected at 620 nm and calculated according to the EB standard curve. The experiments were repeated three times.

### Statistical Analysis

The data are presented as the means ± standard error of mean (SEM). Statistical analysis of data having equal variance was performed by one-way analysis of variance (ANOVA) followed by Tukey's Post Hoc test where appropriate. Quantification of band densities was performed using Image J. The data are presented as the mean ± SEM at 0–8 hours post-LPS treatment. The lung weights observed in the control and LPS groups were compared using Student's t-test. *P<0.05. A value of p<0.01 was considered highly significant. A value of p<0.05 was considered statistically significant.

## Results

### TNF-α Inhibited A549 Cell viability, Increased Paracellular Permeability, HIF-1α Expression and Decreased VASP Expression

**Figure 1:**
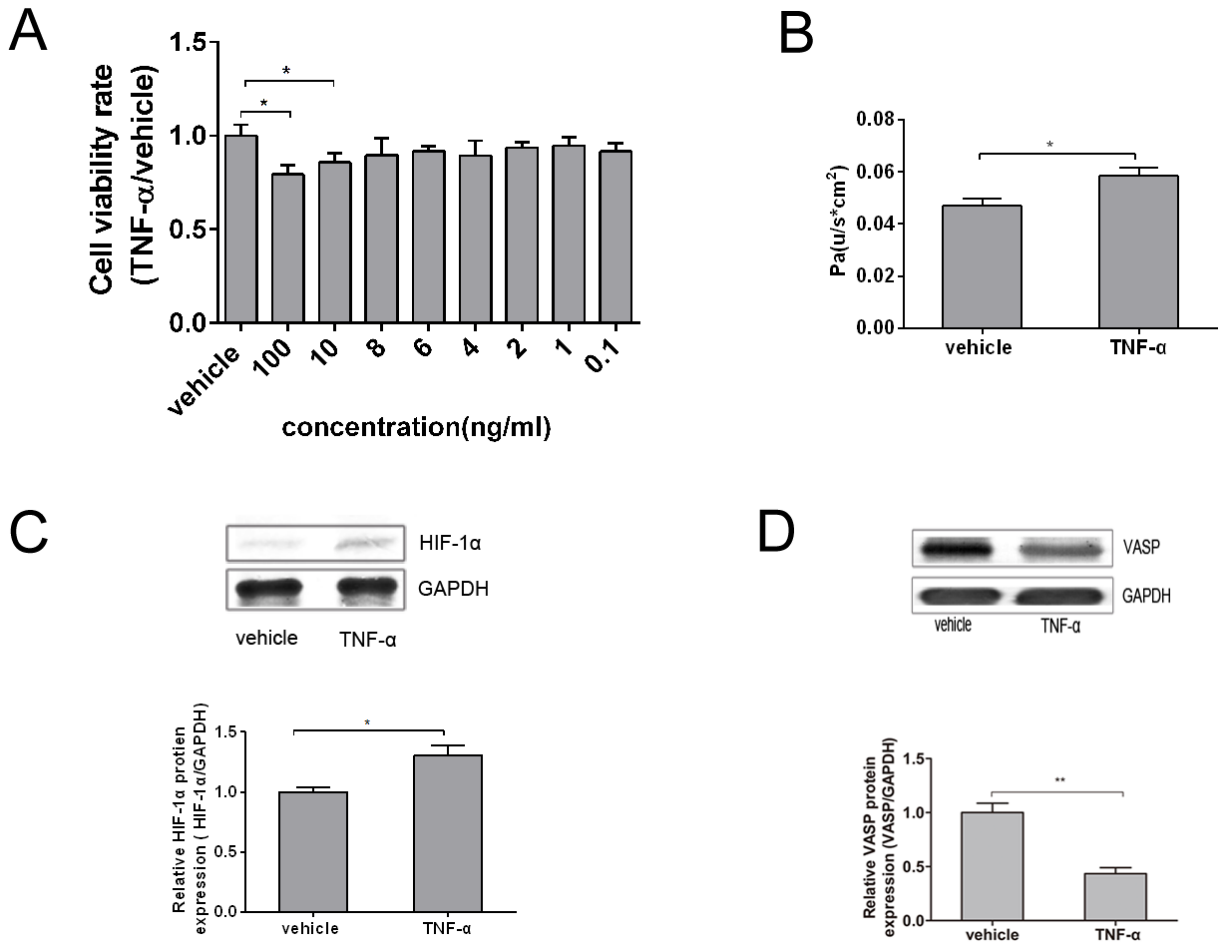
TNF-α inhibited A549 cell viability, increased paracellular permeability, HIF-1α expression and decreased VASP expression. (A) Cells were evenly seeded in 96-well plates at a density of 10^3^–10^4^ cells per well (200 µL per well) and then incubated with TNF-α (0 – 100 ng/mL) for 24 hours. The cell viability rates were examined using an MTT assay, as shown in the bar graph. Compared with 0 ng/mL TNF-α (vehicle control), the stimulation with 0.1–8 ng/mL TNF-α did not affect cell viability (p>0.05). In contrast, 10 ng/mL and 100 ng/mL TNF-α significantly inhibited the viability of A549 cells (*p<0.05) in a dose-dependent manner with reduction rates of 14.3% and 20.7%, respectively. (B) 200 µL of 1×10^5^/mL A549 cell suspension was seeded in the upper chamber of a Transwell that was previously covered with 1% gelatin until 80% confluence. After the well was pre-incubated with 10 ng/mL TNF-α for 24 hours, the Pa was determined according to the rate of fluorescence intensity of FITC-Dextran in the upper and lower chambers by a multifunctional microplate reader, as shown in the bar graph, TNF-α treatment increased Pa by 23.4% compared with the control group. (C) A549 cells were exposed to 10 ng/mL TNF-α for 24 hours, and the total protein of HIF-1α was then extracted. Western Blotting analysis was then used to evaluate the total protein level with each antibody against total HIF-1α, as shown in the bar graph, which represents a graphical representation of the relative density of HIF-1α to GADPH. TNF-α dramatically increased HIF-1α expression by 30.7% compared with the vehicle control. (D) A549 cells were exposed to 10 ng/mL TNF-α for 24 hours, and the total VASP protein was then extracted. Western Blotting analysis was then used to evaluate the total protein level with each antibody against total VASP, as shown in the bar graph. TNF-α inhibited VASP expression by 56.6% compared with the vehicle control. GAPDH served as a loading control; representative blots from three independent experiments with similar results are shown. The relative protein expression levels obtained for HIF-1α or VASP/GAPDH were used in bar graphs C and D. The test was repeated three times with identical results. The data are presented as the mean ± SEM. *p<0.05, **p<0.01 vs. vehicle control.

A MTT assay was used to determine the appropriate concentration of TNF-α that stimulated the viability of A549 cells. The cells were evenly seeded in 96-well plates at a density of 1×10^3^∼1×10^4^/mL cells per well (200 µL per well) and then incubated with TNF-α (0–100 ng/mL) for 24 hours. The absorbance of each well was measured at 570 nm with an ELISA plate reader. As shown in Figure 1A, stimulation with 0.1–8 ng/mL TNF-α did not produce an effect on cell viability compared to the vehicle control (p>0.05). In contrast, 10 ng/mL and 100 ng/mL TNF-α significantly inhibited the viability of A549 cells (p<0.05) in a dose-dependent manner with reduction rates of 14.3% and 20.7%, respectively. We also realized that relatively high TNF-α concentrations may inhibit cell viability, which would reduce the cell density as a direct result of the permeability increase. Thus, choosing an appropriate TNF-α concentration was essential, as it could, on the one hand, impair cell viability, and, on the other hand, avoid cell densities that are too low to maintain the permeability. Taken together, the data lead to the use of 10 ng/mL TNF-α treatment in the subsequent experiments to mimic the impairment effect of TNF-α on A549 cells.

The impairment effect of TNF-α on the permeability of A549 cells was determined via a monolayer cell permeability assay and expressed as Pa. Briefly, 200 µL of a 1×10^3^∼1×10^4^/mL A549 cell suspension was seeded in the upper chamber of a Transwell that was previously covered with 1% gelatin until 80% confluence. After being pre-incubated in 10 ng/mL TNF-α for 24 hours, the Pa was then determined according to rate of fluorescence intensity of FITC-Dextran in the upper and lower chambers as measured using a multifunctional microplate reader. As shown in Figure 1B, TNF-α treatment increased Pa by 23.4% compared with the control group (p<0.05). These data indicate that TNF-α could dramatically increase the paracellular permeability.

In addition to the increase in cell permeability, we also detected a change in the HIF-1α and VASP expression level. After being treated with 10 ng/mL TNF-α for 24 hours, the total protein of the A549 cells was extracted. The HIF-1α and VASP expression levels were then detected by western blotting. As shown in Figure 1C and 1D, TNF-α dramatically promoted HIF-1α expression by 30.7%, and the difference was significant compared with the vehicle control (p<0.05). TNF-α inhibited VASP expression by 56.6%, and the difference was significant compared with the vehicle control (p<0.01).

In conclusion, the results revealed that appropriate TNF-α stimulation could evidently increase the paracellular permeability and HIF-1α expression while decreasing the VASP expression. The following experiments were conducted to further identify the possible relationship between TNF-α, HIF-1α and VASP.

### TNF-α Increased A549 Permeability by Repressing VASP Expression through the Activation of HIF-1α

**Figure 2:**
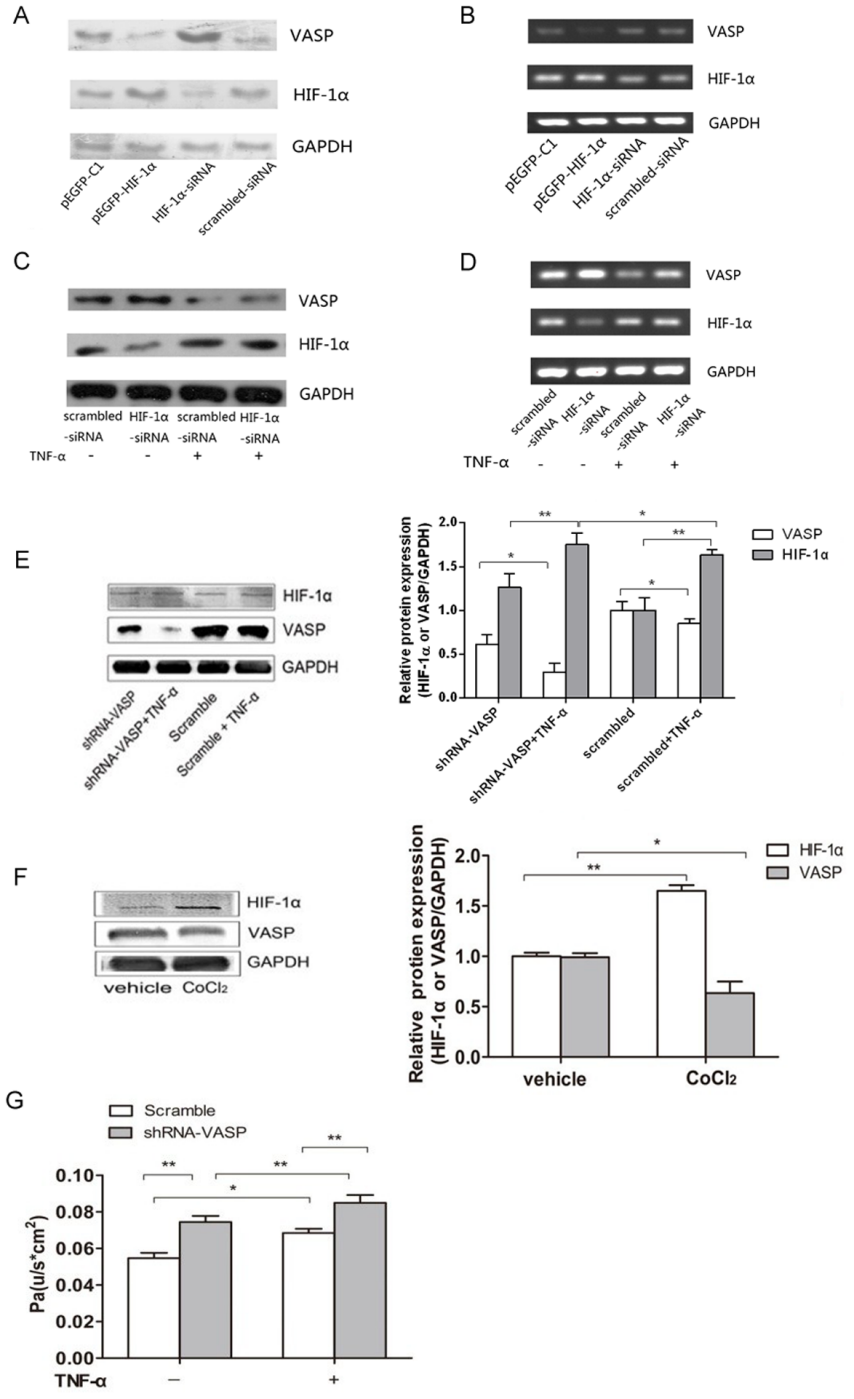
TNF-α increased A549 permeability by repressing VASP expression, through the activation of HIF-1α. (A) A549 cells were transfected with siRNA-HIF-1α or pEGFP-C1-HIF-1α for 24 hours as indicated. Scrambled siRNA and pEGFP-C1 vector were used as controls. The HIF-1α and VASP protein expression levels were then determined by western blotting. GAPDH served as a loading control. (B) A549 cells were transfected with siRNA-HIF-1α or pEGFP-C1-HIF-1α for 24 hours as indicated. HIF-1α and VASP mRNA expression levels were then determined by RT-PCR. Figure 2A and 2B indicate that VASP expression of both mRNA levels and protein levels were repressed by HIF-1α and that VASP expression was dramatically activated by HIF-1α knockdown in A549 cells. In contrast, HIF-1α overexpression led to the decreased VASP expression. (C) Cells were treated with TNF-α after transfection with siRNA-HIF-1α for 24 h. Scrambled siRNA was used as a control. The HIF-1α and VASP protein expression levels were then determined by western blotting. (D) Cells were treated with TNF-α after transfection with siRNA-HIF-1α for 24 h. Scrambled siRNA was used as a control. The HIF-1α and VASP mRNA expression levels were then determined by RT-PCR. Figure 2C and 2D indicate that in the absence of TNF-α, the expression of both HIF-1α and VASP were maintained at moderate protein levels and the knockdown of HIF-1α led to increased VASP expression in A549 cells. Upon stimulation by TNF-α, the level of HIF-1 α was elevated accompanied by a suppression of VASP expression. However, the suppression of VASP by TNF-α could be relieved through HIF-1α knockdown. (E) A549 cells were transfected with shRNA-VASP or scrambled shRNA and then treated with 10 ng/mL TNF-α or untreated for 24 hours as indicated. The HIF-1α and VASP expression levels were then determined by western blotting. In the absence of TNF-α, VASP expression in the shRNA-VASP group decreased by 15.6% versus the scrambled group (**p<0.01). VASP expression in the shRNA-VASP+TNF-α group was 70.4% lower than that in the scrambled+TNF-α group (*p<0.05). The transfection of shRNA-VASP did not affect the HIF-1α expression level (scrambled vs. shRNA-VASP, p>0.05; scrambled+TNF-α vs. shRNA-VASP+TNF-α, p>0.05). Conversely, TNF-α stimulation dramatically elevated HIF-1α expression (scrambled vs. scrambled+TNF-α, **p<0.01; shRNA-VASP vs. shRNA-VASP+TNF-α, *p<0.05). (F) A549 cells were exposed to 200 µg/mL CoCl_2_ or vehicle for 24 hours. The HIF-1α and VASP expression levels were then detected via western blotting. HIF-1α expression increased by 64.9% in the CoCl_2_ group compared with the vehicle group (**p<0.01), whereas VASP expression decreased by 36.7% in the CoCl_2_ group compared with the vehicle group (*p<0.05). GAPDH served as a loading control. Representative blots from three independent experiments with similar results are shown. The relative protein expression levels obtained for HIF-1α or VASP/GAPDH were used in bar graphs B and C. (G) A549 cells were transfected with shRNA-VASP or scrambled shRNA and then treated with 10 ng/mL TNF-α or untreated for 24 hours as indicated. The paracellular permeability was detected via a monolayer paracellular cells permeability assay and expressed as Pa. The Pa in the shRNA-VASP group increased significantly compared with the scrambled group, 36.0% in the absence of TNF-α (**p<0.01) and 24.3% in the presence of TNF-α (**p<0.01). The Pa in the shRNA-VASP+TNF-α group was 14.2% higher than that in the shRNA-VASP group (**p<0.01). In addition, TNF-α induced a 25.0% increase in Pa (*p<0.05) between the two scrambled groups. The test was repeated three times with identical results. The data are presented as the mean ± SEM. *p<0.05, **p<0.01.

The preliminary experiments demonstrated both dramatically higher HIF-1α and lower VASP expression levels in TNF-α-treated A549 cells compared with the control group cells. To detect the specific relationship between TNF-α,HIF-1α and VASP, first, we transfected A549 cells with siRNA-HIF-1α to inhibit HIF-1α expression to investigate the relationship between HIF-1α and VASP. A549 cells were transfected with siRNA scrambled as a control. We observed that when transfected with HIF-1α-siRNA, which knocked down HIF-1α expression, VASP expression at the mRNA level and protein level was significantly increased compared with the control group. In contrast, while transfected with pEGFP-Cl-HIF-1α to overexpress HIF-1α expression, VASP mRNA level and protein level were significantly decreased compared with the control group. These complementary results indicated that HIF-1α was strongly involved in the adjustment of VASP expression both at the transcription and protein level (Figure 2A and 2B). To further investigate whether HIF-1α was strongly involved in TNF-α-induced suppression of VASP, we explored the potential role of HIF-1α in the presence or the absence of TNF-α treatment. We transfected A549 cells with HIF-1α-siRNA and then treated them with or without 10 ng/ml TNF-α (which was proven to exert the most effective impairment effects on A549 cells). Western blotting was used to detect HIF-1α and VASP protein expression level. RT-PCR was applied to determine the mRNA expression level in A549 cells. As shown in Figure 2C and 2D, HIF-1α-siRNA, which had a knockdown effect on HIF-1α expression, resulted in the decrease in HIF expression and the increase in VASP expression both at the protein level and mRNA level. Upon stimulation with TNF-α, HIF-1α expression was elevated, and the VASP expression level was suppressed in parallel. However, we found that such an inhibitory effect was relieved in the HIF-1α knockdown group. Thus, the results indicated that HIF-1α mediated the TNF-α induced suppression of VASP.

In addition, to investigate the relationship between VASP and HIF-1α, we transfected A549 cells with shRNA-VASP (pcDNA6.2-GW/EmGFP MIR-VASP) to inhibit VASP expression. A549 cells were transfected with scrambled shRNA (pcDNA6.2-GW/EmGFP MIR) as a control. After having been treated with or without TNF-α for 24 hours, the HIF-1α and VASP expression levels in A549 cells were then determined by western blotting. As shown in Figure 2E, VASP expression in the shRNA-VASP group decreased by 15.6% compared with the scrambled group in the absence of TNF-α (p<0.01), which suggests that shRNA-VASP was successfully transfected and exerted an obvious inhibitory effect on VASP expression. Moreover, we further explored the potential role of VASP in the presence or the absence of TNF-α treatment. VASP expression in the shRNA-VASP group was 70.4% lower than that in the scrambled group in the presence of TNF-α (p<0.01). The difference in the two inhibition rates (70.4% vs. 36.6%) indicates that the combination of shRNA-VASP and TNF-α could better inhibit VASP expression than the single factor shRNA-VASP, which further indicates that TNF-α could also inhibit VASP expression. To explore the potential mechanism by which TNF-α inhibited VASP expression, we also determined the expression level of the transcription factor HIF-1α in this experiment. The HIF-1α expression level was not significantly different in shRNA-VASP group from the control group both in the absence and presence of TNF-α (p>0.05). This lack of difference could be interpreted as the downregulation of VASP not inducing changes in HIF-1α, which indicates that HIF-1α could not act downstream of VASP to increase the paracellular permeability. In addition, the single factor of TNF-α stimulation dramatically elevated HIF-1α expression, as compared with the respective TNF-α (-) groups (without TNF-α stimulation) both in the shRNA-VASP group (p<0.05) and scrambled group (p<0.01) with increases of 72.2% and 70.4%, respectively, as shown in Figure 2E.

To further investigate the relationship between HIF-1α and VASP, we utilized cobalt chloride (CoCl_2_) to mimic a hypoxic environment, which activates HIF-1α. A549 cells were exposed to 200 µg/mL CoCl_2_ for 24 hours, and the HIF-1α and VASP expression levels were then detected via western blotting. As shown in Figure 2F, HIF-1α expression increased by 64.9% compared with the vehicle group when exposed A549 cells were exposed to a hypoxic environment (p<0.01), whereas VASP expression decreased by 36.7% (p<0.05). Taken together with the data in Figure 2E, these findings indicate that HIF-1α could not act downstream of VASP. Thus, we concluded that VASP downregulation acted downstream of HIF-1α activation when increasing the paracellular permeability. This finding became more evident when considering the article by Ke Su, another member in our team, who found that HIF-1α modulated the expression of VASP by directly binding to the VASP promoter. In addition, Peter Rosenberger's recent study also demonstrated evidence of a TNF-α/HIF-1α/VASP pathway by elucidating that TNF-α could activate HIF and subsequently inhibit VASP expression in HMEC-1 in hypoxia-induced pneumonic edema in mice.

To further study the direct effect of VASP downregulation on the paracellular permeability, we transfected A549 cells with shRNA-VASP to inhibit VASP expression; A549 cells were also transfected with scrambled shRNA as a control. The cells were then treated with or without TNF-α. The paracellular permeability was assessed by a monolayer paracellular cell permeability assay. As shown in Figure 2G, Pa increased significantly by 36.0% in the absence of TNF-α (p<0.01) and 24.3% in the presence of TNF-α (p<0.01) in the shRNA-VASP group compared with the scrambled group, which elucidated that the repression of VASP expression increased the paracellular permeability. Furthermore, the combination of shRNA-VASP and TNF-α treatment increased Pa more than the single factor of shRNA-VASP; Pa was 14.2% higher in the shRNA-VASP+TNF-α group than in the shRNA-VASP group (p<0.01). In addition, we found that treatment with TNF-α induced a 25.0% increase of Pa between the two scrambled groups (p<0.05).

Collectively, the data in Figure 2 demonstrate that TNF-α mediated an increase in HIF-1α, which then downregulated VASP expression and finally led to an increase in the paracellular permeability of A549 cells.

### LPS-Induced Acute Lung Injury Model via Repressing Lung Barrier Function in Balb/c

**Figure 3:**
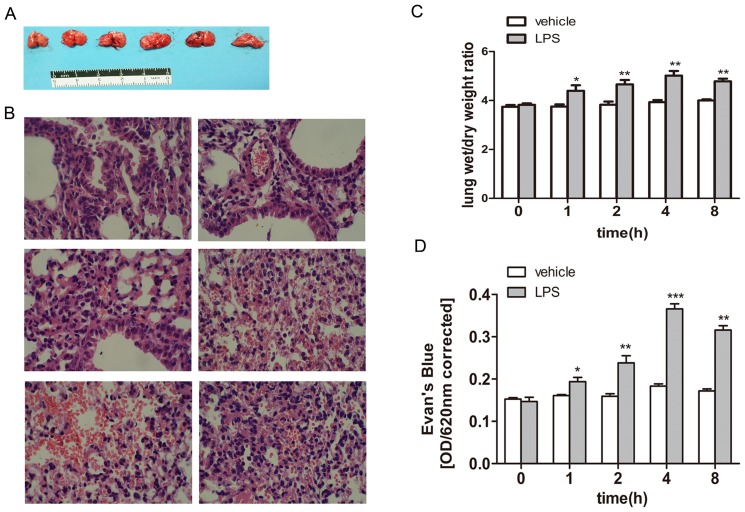
LPS induced acute lung injury model via repressing lung barrier function in Balb/c(×400). (A) 10 Balb/c mice were randomly divided into two groups with five mice per group. The mice in the experimental group were peritoneally injected with 0.01 mg/g LPS fluid per mouse to model ALI, whereas the mice in the control group were injected with the same dose of saline. After 0–8 hours of stimulation, the left lung tissues of each mouse were carefully separated, as shown at the macroscopic level. (B) The specimens were then fixed in 4% paraformaldehyde for HE staining, as shown at the microscopic level. Images taken at ×400 (G) (C) after LPS induction, and all right lung tissues were separated and placed on filter paper to measure their wet weight. The tissues were then placed in a 60°C thermotank for 24 hours to obtain the dry weight. The W/D (weight/dry) data are shown in the bar graph. Compared with the 0 hour group (control group), the W/D value increased significantly by 17.1%, 21.4% and 27.5% at 1 hour, 2 hours and 4 hours, respectively. However, the absolute W/D value began to decrease at 8 hours compared with the 4-hour value, but this difference was insignificant. (D) After LPS induction, the lung barrier function was assayed using the Evans blue dye extra-barrier technique to determine the integrity of the alveolar epithelium integrity. Balb/c mice were injected with 0.01 mg/g Evans Blue per mouse in the tail vein 30 minutes before execution. The absorbance of Evans Blue was detected in an ultraviolet spectrophotometer at 620 nm, as shown in the bar graph. The absorbance value began to increase at 1 hour compared with the vehicle group. The value increased over time. The absorbance value reached its peak at 4 hours and decreased somewhat at 8 hours compared with the 4-hour value, but the difference was not significant. The test was repeated three times with identical results. The data are presented as the mean ± SEM. *p<0.05, **p<0.01, ***p<0.001 vs. vehicle control.

Balb/c mice were randomly and equally divided into two groups. The mice in the in experimental group were peritoneally injected with 0.01 mg/g LPS fluid per mouse to model ALI, whereas mice in the control group were injected with the same dose of saline. After 0–8 hours of stimulation, the left lung tissues of each mouse were carefully separated and then fixed in 4% paraformaldehyde for HE staining or homogenized in PBS for an enzyme-linked immunosorbent assay (ELISA) assay. The results obtained from the macroscopic specimens are shown in Figure 3A; the lung tissues of Balb/c mice began to display acute lung injury after 1 h compared with the 0 hour group. To be specific, the macroscopic lung tissues of the control group and 0 hour clearly appeared pink and expanded equally and satisfactorily without any congestion, edema or infarction. However, the lungs of the LPS-induced groups exhibited kermesinus, were larger in size and displayed mutic margins with obvious edema. Edema and congestion became particularly evident at 4 hours, when some lungs even displayed a pink, frothy fluid.

We used HE staining to further precisely investigate the changes in the microscopic level after LPS induction. The microscopic structures of lung tissues (400×) are shown in Figure 3B. Alveoli demonstrated structural integrity in the 0 hour group. The alveolar septa were consistent. The alveolar walls were thin, and the lungs displayed little inflammatory infiltration and effusion. However, the 1-hour group and other LPS-induced groups demonstrated diverse degrees of enlargement and congestion in the pulmonary capillaries. In addition, the alveolar septum was incrassated, with inflammatory cells infiltrating the alveoli and interstitial tissue. The above changes also varied as the time of stimulation proceeded. The most severe point was 4 hours, when a hemorrhage region began to appear. At 8 hours, the effusion of red blood cells receded a bit, with inflammatory cells increasing compared with the 4-hour group.

After LPS induction, all right lung tissues were separated and placed on filter paper to measure their wet weight. The tissues were then placed in a 60°C thermotank for 24 hours to obtain the dry weight. The W/D (weight/dry) data are shown in Figure 3C; the W/D value was 4.39±0.12 at 1 hour. This value was higher than that of the control group, and the difference was significant (p<0.05). The differences became more pronounced over time. Specifically, the W/D value increased approximately 17.1%, 21.4% and 27.5% at 1 hour, 2 hours and 4 hours, respectively. However, the W/D absolute value began to decrease at 8 hours compared with 4 hours, but this difference was not significant.

After LPS induction, the lung barrier function was assayed using the Evans blue dye extra-barrier technique to determine the alveolar epithelium integrity. Balb/c mice were injected with 0.01 mg/g Evans Blue per mouse in the tail vein 30 minutes before sacrifice. The absorbance of Evans Blue was detected with an ultraviolet spectrophotometer at 620 nm. As shown in Figure 3D, the absorbance value began to increase at 1 hour. This value was 0.19±0.02, which was an increase of approximately 20.4% compared with the vehicle group, and the difference was significant (p<0.05). The value changed over time. Specifically, the absorbance values were 0.24±0.02, 0.37±0.04 and 0.32±0.06 at 2 hours, 4 hours and 8 hours, respectively. The value clearly increased, and the differences were significant compared with the vehicle group. The absorbance value reached its peak at 4 hours and somewhat decreased at 8 hours compared with the 4-hour value, but the difference was insignificant.

The experiments demonstrated that LPS successfully induced acute lung injury via repressing lung barrier function. We verified its success not only at the macroscopic level but also at the microscopic and statistic level. Moreover, lung injury was most severe at 4 hours and 8 hours in the given experimental conditions.

### VASP Was Downregulated through TNF-α-Induced Activation of HIF-1α during Acute Lung Injury in Vivo

**Figure 4:**
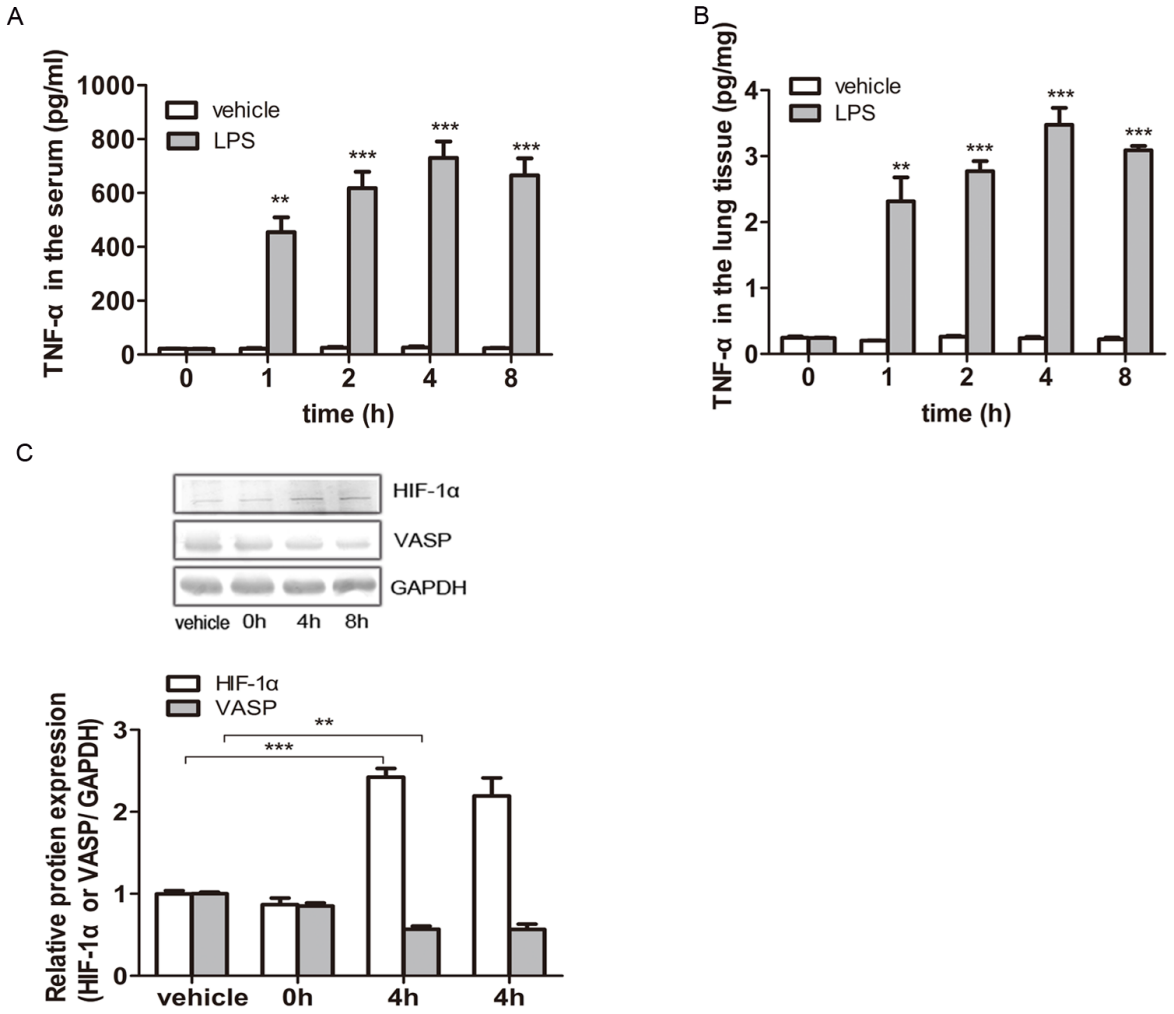
VASP was downregulated via the TNF-α-induced activation of HIF-1α during acute lung injury in vivo. Balb/c mice were peritoneally injected with 0.01 mg/g LPS to model ALI or the same dose of saline in the vehicle group. The animals were treated for 0–8 hours. (A) After 0–8 hours of stimulation, the sera of the animal models were prepared via orbital blood collection, and ELISA was performed to detect the concentration of TNF-α in the serum. In the vehicle groups, the TNF-α concentration in the serum remained at a very low level (<30 pg/mL) and displayed no obvious changes over time (p>0.05 vs. vehicle). In contrast, the sera from mice treated with LPS for 0–8 hours all displayed much higher TNF-α concentrations (>450 pg/mL), which peaked at 4 hours (>700 pg/mL), compared with the vehicle groups (**p<0.01 and ***p<0.001 vs. vehicle). (B) After LPS treatment, the supernatant liquid of lung tissues was prepared from the excised left lungs, and ELISA was performed to detect the concentration of TNF-α in the lung tissues. The TNF-α concentration in the lung tissues also remained at a very low level (<0.3 pg/mg) in the vehicle groups and displayed no obvious changes over time (p>0.05 vs. vehicle). Conversely, the TNF-α concentration in the lung tissues from LPS-induced ALI mice was dramatically higher than that of the vehicle groups (>2.0 pg/mg; **p<0.01 and ***p<0.001 vs. vehicle) and peaked at 4 hours (>3.4 pg/mg). (C) The HIF-1α and VASP expression levels in mouse lung tissues were detected by western blotting. GAPDH served as a loading control. Representative blots from three independent experiments with similar results are shown. The relative protein expression levels obtained for HIF-1α or VASP/GAPDH are shown in the bar graphs. Compared with the vehicle group, the HIF-1α levels in the lung tissues were evidently elevated by 142.3% in the 4-hour group (***p<0.001) and 119.3% in the 8-hour group (**p<0.01 vs. vehicle). Correspondingly, VASP expression decreased by 43.1% in the 4-hour group (**p<0.01 vs. vehicle) and 43.5% in the 8-hour group (**p<0.01 vs. vehicle). The test was repeated three times with identical results. The data are presented as the mean ± SEM. *p<0.05, **p<0.01, ***p<0.001.

After LPS treatment, the serum of animal models was prepared via orbital blood collection, and the supernatant liquid of lung tissues was obtained from the excised left lungs. ELISA was adopted to detect the concentration of TNF-α in both the serum and lung tissues. As shown in Figure 4A, the TNF-α concentration in the serum remained at a very low level (<30 pg/mL) in the vehicle groups (with saline injection) and displayed no obvious changes over time (p>0.05). In contrast, the serum from mice treated with LPS for a total of 1 hour, 2 hours, 4 hours or 8 hours all displayed a much higher TNF-α concentration (>450 pg/mL), which peaked at 4 hours (>700 pg/mL), compared with the vehicle groups (1 hour: p<0.01; 2, 4, or 8 hours: p<0.001).

As shown in Figure 4B, the TNF-α concentration in the lung tissues also remained at a very low level (<0.3 pg/mg) in the vehicle groups (with saline injection) and displayed no obvious changes over time (p>0.05). Conversely, the TNF-α concentration in the lung tissues from LPS-induced ALI mice was dramatically higher than that of the vehicle groups (>2.0 pg/mg; 1 hour: p<0.01; 2, 4, or 8 hours: p<0.001) and peaked at 4 hours (>3.4 pg/mg). Taken together, we also found that changes in the TNF-α concentration created by different time courses of LPS treatment were synchronized in both the serum and lung tissues.

Next, we detected the HIF-1α and VASP expression levels in mouse lung tissues by western blotting to assess the TNF-α/HIF-1α/VASP pathway in vivo. As shown in Figure 4C, the HIF-1α and VASP expression levels in the lung tissues from mice treated with LPS for 0 hours did not differ significantly from the vehicle group (p>0.05). However, HIF-1α expression was evidently elevated by 142.3% (p<0.001) and 119.3% (p<0.01) compared with the control group in the lung tissues from mice treated with LPS for 4 hours and 8 hours, respectively. Correspondingly, VASP expression decreased by 43.1% in the 4-hour group (p<0.01) and by 43.5% in the 8-hour group (p<0.01).

Combined with the results obtained in the cellular experiments, we demonstrated that VASP was downregulated in vivo via the TNF-α-induced activation of HIF-1α during acute lung injury.

## Discussion

The release of cytokines or proinflammatory factors during the systemic inflammation in ALI injures pulmonary vascular endothelial cells and alveolar epithelial cells, which increases the permeability of the pulmonary capillary membrane. Consequently, liquid protein permeates from the vessel lumen into the interstitial space and alveolus, followed by external respiratory dysfunction, which subsequently leads to a lethal abnormal decrease of the arterial partial pressure of oxygen. ALI occurs in serious conditions, such as severe pancreatitis, massive transfusion, sepsis and lung injuries, which cause respiratory distress, diffuse lung infiltrates and respiratory failure. The total incidences of ARF, ALI and ARDS in Sweden, Denmark and Iceland are 77.6, 17.9 and 13.5 patients per 100,000/yr, respectively. These figures were reported by Luhr OR in 1999 and associated with a significantly higher mortality [23]. In addition, he reported that the ninety-day mortality was 41.0% for ARF, which included ALI and ARDS patients reported on in the *American Journal of Respiratory and Critical Care Medicine* [24]. Thus, research on the hyperpermeability of the alveolar-capillary membrane induced by cytokines or proinflammatory factors in ALI is significant and vital for guiding medical treatment decisions.

The alveolar-capillary membrane exists in the gas-exchanging region of the lungs. It consists of type I pneumocytes of the alveolar wall, the endothelial cells of the capillaries and the basement membrane between the two cells. The blood-gas barrier is extremely thin to allow sufficient oxygen diffusion; however, it is extremely strong. This strength is mainly attributable to the tight junctions between neighboring endothelial and epithelial cells. These junctions depend on actin kinetics, which are regulated by actin-associated proteins [25]. As a core member of actin-associated proteins, VASP participates in various modulations of the morphology and functions of cells via the rearrangement of the cytoskeleton, such as migration, polarity, conglutination, shrinkage, movement, etc. [26], [27]. Emerging evidence has revealed that VASP may also play a key role in maintaining the function of physiological barriers [28]. For example, VASP affects intercellular adhesion via the α-catenin-dependent promotion of actin reorganization and polymerization, which merges punctae into a single row and seals cell borders. Furthermore, the suppression of VASP can result in extensive edema and hemorrhage in tissues by impairing the contraction of actin and inducing an abnormal response to shear stress [29]. Recently, Henes J's study indicated that TNF-α could activate HIF-1α and subsequently inhibit VASP expression in HMEC-1 in hypoxia-induced pneumonic edema in mice [30]. Stamatina's study demonstrated that TNF-α induces expression of HIF-1α mRNA and protein in airway smooth muscle cells [31]. In addition, Xia L's study indicates that TNF-α leads to the stabilization of HIF-1α, which then translocates into nucleus and binds with the target gene promoter [32]. Thus, we sought to determine whether cytokines could mediate the development of pneumonic edema by impairing tight junctions in both endothelial cells and type I pneumocytes during LPS-induced ALI. In recent studies, LPS has been widely used to induce systemic inflammation [33]. It has also been reported that LPS induces expression of numerous pro-inflammatory cytokines, including TNF-α, interleukin-6 (IL-6), and interleukin-1(IL-1), which were mainly released by macrophage (Mφ) [34]. In acute lung injury, especially in LPS-induced lung inflammation, TNF-α plays a dominant role in LPS-induced mouse mortality [35], [36]. Thus, to study the role TNF-α plays in ALI, we employed LPS-induced ALI models. Here, we observed TNF-α increased significantly at the serum and organismal levels in LPS-induced ALI mice, which was accompanied by hyperpermeability of the alveolar-capillary membrane as well as the downregulation of VASP expression [37]. In addition, we demonstrated that a low concentration (10 ng/mL) of TNF-α could induce HIF-1α upregulation and subsequently dramatically decrease VASP expression in cultured type II alveolar A549 cells in vitro. Thus, HIF-1α-dependent VASP regulation likely contributed, at least in part, to a change in the alveolar-capillary barrier permeability in LPS-induced ALI via enhanced TNF-α secretion.

Inflammatory cytokines participate in wide ranges of biological effects, such as the innate immune response, the adaptive immune response during inflammation and inflammation-related diseases. The majority of these functions are achieved via the activation of a signaling cascade by releasing diverse growth factors, such as VEGF and PDEGF, and transcription factors, such as NF-κB and HIF-1α [38], [39]. The related research by Janek Henes's indicates that NF-κB activation accelerates the increase of alveolar-capillary permeability during TNF-α-induced pneumonia in VASP knockout mice [30]. Furthermore, we previously identified that HIF-1α could directly inhibit VASP transcription during the TNF-α-induced metastasis and adhesion of breast cancer MCF-7 cells [15]. In this study, we determined that HIF-1α expression in lung tissues increased in LPS-induced ALI mice, whereas TNF-α secretion in lung tissues and serum increased significantly; furthermore, 10 ng/mL TNF-α could promote HIF-1α expression at the protein level in cultured type II alveolar A549 cells. In addition, this treatment inhibited VASP expression and impaired the formation of alveolar-alveolar tight junctions. Conversely, pretreating A549 cells with specific VASP-siRNA could rescue VASP expression and repair the tight junction impairment caused by 10 ng/mL TNF-α. These results indicate that HIF-1α was likely the key mediator of the TNF-α-induced impairment of alveolar-capillary barrier in LPS-reduced ALI.

VASP plays an essential role in mediating stress fiber formation and is involved in membrane ruffling and aggregation [40], [41]. The protein has three regions: the EVH1 region at the amino terminal, the EVH2 region at the carboxyl terminal and the proline-rich region (PRR) in the middle. The EVH2 region lengthens F-actin by promoting G-actin's connection with F-actin's assembling terminal, and the PRR region can combine with proteins that contain a SH3 motif [27], [42]. ZO-1 contains a SH3 motif and plays a critical role in tight junction complex assembly. The modification of ZO-1 expression and localization leads to increased paracellular permeability. More importantly, VASP colocalizes with ZO-1 at tight junctions to maintain endothelial barrier function [43]. Inflammation cytokines are thought to influence the properties of the alveolar-capillary barrier by actively reorganizing the cytoskeleton [44]. In our experiment, we found that the pulmonary permeability increased at the animal level, whereas VASP expression decreased. The same changes were observed in excised alveolar epithelial cells. These results demonstrated that VASP played a key role in establishing and maintaining the barrier function as a major actin binding protein.

Taken together, our data demonstrate that VASP, which is negatively regulated by TNF-α, induces HIF-1α activation during the acute pulmonary inflammation process and plays an important role in the impairment of the alveolar-capillary barrier. These findings are significant because efforts to understand better the hidden beneficial role of VASP in the blood gas barrier function facilitate medical therapy for ALI treatment.

## References

[1] Dushianthan A, Grocott MP, Postle AD, Cusack R (2011) Acute respiratory distress syndrome and acute lung injury. Postgrad Med J 87: 612–622. 21642654 10.1136/pgmj.2011.118398

[2] Patel BV, Wilson MR, O'Dea KP, Takata M (2013) TNF-induced death signaling triggers alveolar epithelial dysfunction in acute lung injury. J Immunol 190: 4274–4282. 23487422 10.4049/jimmunol.1202437 PMC3701855

[3] Miyoshi K, Yanagi S, Kawahara K, Nishio M, Tsubouchi H, et al (2013) Epithelial Pten controls acute lung injury and fibrosis by regulating alveolar epithelial cell integrity. Am J Respir Crit Care Med 187: 262–275. 23239155 10.1164/rccm.201205-0851OC

[4] Cornelio Favarin D, Martins Teixeira M, Lemos de Andrade E, de Freitas Alves C, Lazo Chica JE, et al (2013) Anti-inflammatory effects of ellagic acid on acute lung injury induced by acid in mice. Mediators Inflamm 2013: 164202. 23533300 10.1155/2013/164202 PMC3600201

[5] Jin Y, Yu G, Peng P, Zhang Y, Xin X (2013) Down-regulated expression of AQP5 on lung in rat DIC model induced by LPS and its effect on the development of pulmonary edema. Pulm Pharmacol Ther 26: 661–665. 23538169 10.1016/j.pupt.2013.03.013

[6] Ozaki H, Ishii K, Horiuchi H, Arai H, Kawamoto T, et al (1999) Cutting edge: combined treatment of TNF-alpha and IFN-gamma causes redistribution of junctional adhesion molecule in human endothelial cells. J Immunol 163: 553–557. 10395639

[7] Goetze S, Xi XP, Kawano Y, Kawano H, Fleck E, et al (1999) TNF-alpha-induced migration of vascular smooth muscle cells is MAPK dependent. Hypertension 33: 183–189. 9931102 10.1161/01.hyp.33.1.183

[8] Blouin CC, Page EL, Soucy GM, Richard DE (2004) Hypoxic gene activation by lipopolysaccharide in macrophages: implication of hypoxia-inducible factor 1alpha. Blood 103: 1124–1130. 14525767 10.1182/blood-2003-07-2427

[9] Haddad JJ, Land SC (2001) A non-hypoxic, ROS-sensitive pathway mediates TNF-alpha-dependent regulation of HIF-1alpha. FEBS Lett 505: 269–274. 11566189 10.1016/s0014-5793(01)02833-2

[10] Imtiyaz HZ, Simon MC (2010) Hypoxia-inducible factors as essential regulators of inflammation. Curr Top Microbiol Immunol 345: 105–120. 20517715 10.1007/82_2010_74 PMC3144567

[11] Jung YJ, Isaacs JS, Lee S, Trepel J, Neckers L (2003) IL-1beta-mediated up-regulation of HIF-1alpha via an NFkappaB/COX-2 pathway identifies HIF-1 as a critical link between inflammation and oncogenesis. FASEB J 17: 2115–2117. 12958148 10.1096/fj.03-0329fje

[12] Semenza GL (2001) HIF-1, O(2), and the 3 PHDs: how animal cells signal hypoxia to the nucleus. Cell 107: 1–3. 11595178 10.1016/s0092-8674(01)00518-9

[13] Ebert BL, Bunn HF (1998) Regulation of transcription by hypoxia requires a multiprotein complex that includes hypoxia-inducible factor 1, an adjacent transcription factor, and p300/CREB binding protein. Mol Cell Biol 18: 4089–4096. 9632793 10.1128/mcb.18.7.4089 PMC108993

[14] Rosenberger P, Khoury J, Kong T, Weissmuller T, Robinson AM, et al (2007) Identification of vasodilator-stimulated phosphoprotein (VASP) as an HIF-regulated tissue permeability factor during hypoxia. FASEB J 21: 2613–2621. 17412998 10.1096/fj.06-8004com PMC4049288

[15] Su K, Tian Y, Wang J, Shi W, Luo D, et al (2012) HIF-1alpha acts downstream of TNF-alpha to inhibit vasodilator-stimulated phosphoprotein expression and modulates the adhesion and proliferation of breast cancer cells. DNA Cell Biol 31: 1078–1087. 22320863 10.1089/dna.2011.1563

[16] Simon AM, McWhorter AR, Chen H, Jackson CL, Ouellette Y (2004) Decreased intercellular communication and connexin expression in mouse aortic endothelium during lipopolysaccharide-induced inflammation. J Vasc Res 41: 323–333. 15249738 10.1159/000079614

[17] Lidington D, Tyml K, Ouellette Y (2002) Lipopolysaccharide-induced reductions in cellular coupling correlate with tyrosine phosphorylation of connexin 43. J Cell Physiol 193: 373–379. 12384989 10.1002/jcp.10179

[18] Tonon R, D'Andrea P (2002) The functional expression of connexin 43 in articular chondrocytes is increased by interleukin 1beta: evidence for a Ca2+-dependent mechanism. Biorheology 39: 153–160. 12082278

[19] Figueroa XF, Alvina K, Martinez AD, Garces G, Rosemblatt M, et al (2004) Histamine reduces gap junctional communication of human tonsil high endothelial cells in culture. Microvasc Res 68: 247–257. 15501244 10.1016/j.mvr.2004.06.009

[20] Zhao X, Zmijewski JW, Lorne E, Liu G, Park YJ, et al (2008) Activation of AMPK attenuates neutrophil proinflammatory activity and decreases the severity of acute lung injury. Am J Physiol Lung Cell Mol Physiol 295: L497–504. 18586954 10.1152/ajplung.90210.2008 PMC2536800

[21] Abraham E, Carmody A, Shenkar R, Arcaroli J (2000) Neutrophils as early immunologic effectors in hemorrhage- or endotoxemia-induced acute lung injury. Am J Physiol Lung Cell Mol Physiol 279: L1137–1145. 11076804 10.1152/ajplung.2000.279.6.L1137

[22] Wang L, Taneja R, Wang W, Yao LJ, Veldhuizen RA, et al (2013) Human alveolar epithelial cells attenuate pulmonary microvascular endothelial cell permeability under septic conditions. PLoS One 8: e55311. 23393568 10.1371/journal.pone.0055311 PMC3564849

[23] Luhr OR, Antonsen K, Karlsson M, Aardal S, Thorsteinsson A, et al (1999) Incidence and mortality after acute respiratory failure and acute respiratory distress syndrome in Sweden, Denmark, and Iceland. The ARF Study Group. Am J Respir Crit Care Med 159: 1849–1861. 10351930 10.1164/ajrccm.159.6.9808136

[24] Ware LB, Matthay MA (2000) The acute respiratory distress syndrome. N Engl J Med 342: 1334–1349. 10793167 10.1056/NEJM200005043421806

[25] Hohenstein B, Kasperek L, Kobelt DJ, Daniel C, Gambaryan S, et al (2005) Vasodilator-stimulated phosphoprotein-deficient mice demonstrate increased platelet activation but improved renal endothelial preservation and regeneration in passive nephrotoxic nephritis. J Am Soc Nephrol 16: 986–996. 15743999 10.1681/ASN.2004070591

[26] Garcia Arguinzonis MI, Galler AB, Walter U, Reinhard M, Simm A (2002) Increased spreading, Rac/p21-activated kinase (PAK) activity, and compromised cell motility in cells deficient in vasodilator-stimulated phosphoprotein (VASP). J Biol Chem 277: 45604–45610. 12055190 10.1074/jbc.M202873200

[27] Krause M, Dent EW, Bear JE, Loureiro JJ, Gertler FB (2003) Ena/VASP proteins: regulators of the actin cytoskeleton and cell migration. Annu Rev Cell Dev Biol 19: 541–564. 14570581 10.1146/annurev.cellbio.19.050103.103356

[28] Vasioukhin V, Bauer C, Yin M, Fuchs E (2000) Directed actin polymerization is the driving force for epithelial cell-cell adhesion. Cell 100: 209–219. 10660044 10.1016/s0092-8674(00)81559-7

[29] Furman C, Sieminski AL, Kwiatkowski AV, Rubinson DA, Vasile E, et al (2007) Ena/VASP is required for endothelial barrier function in vivo. J Cell Biol 179: 761–775. 17998398 10.1083/jcb.200705002 PMC2080895

[30] Henes J, Schmit MA, Morote-Garcia JC, Mirakaj V, Kohler D, et al (2009) Inflammation-associated repression of vasodilator-stimulated phosphoprotein (VASP) reduces alveolar-capillary barrier function during acute lung injury. FASEB J 23: 4244–4255. 19690214 10.1096/fj.09-138693 PMC2812050

[31] Tsapournioti S, Mylonis I, Hatziefthimiou A, Ioannou MG, Stamatiou R, et al (2013) TNFalpha induces expression of HIF-1alpha mRNA and protein but inhibits hypoxic stimulation of HIF-1 transcriptional activity in airway smooth muscle cells. J Cell Physiol 228: 1745–1753. 23359428 10.1002/jcp.24331

[32] Xia L, Mo P, Huang W, Zhang L, Wang Y, et al (2012) The TNF-alpha/ROS/HIF-1-induced upregulation of FoxMI expression promotes HCC proliferation and resistance to apoptosis. Carcinogenesis 33: 2250–2259. 22831955 10.1093/carcin/bgs249

[33] Li Y, Dong JB, Wu MP (2008) Human ApoA-I overexpression diminishes LPS-induced systemic inflammation and multiple organ damage in mice. Eur J Pharmacol 590: 417–422. 18593575 10.1016/j.ejphar.2008.06.047

[34] Ato M, Iwabuchi K, Shimada S, Mukaida N, Onoe K (2002) Augmented expression of tumour necrosis factor-alpha induced by lipopolysaccharide in spleen of human monocyte chemoattractant protein-1 transgenic mouse enhances the lipopolysaccharide sensitivity of the marginal zone macrophages. Immunology 106: 554–563. 12153519 10.1046/j.1365-2567.2002.01450.x PMC1782746

[35] Kim JH, Kim SJ, Lee IS, Lee MS, Uematsu S, et al (2009) Bacterial endotoxin induces the release of high mobility group box 1 via the IFN-beta signaling pathway. J Immunol 182: 2458–2466. 19201901 10.4049/jimmunol.0801364

[36] Freedman SD, Weinstein D, Blanco PG, Martinez-Clark P, Urman S, et al (2002) (1985) Characterization of LPS-induced lung inflammation in cftr-/- mice and the effect of docosahexaenoic acid. J Appl Physiol 92: 2169–2176. 11960971 10.1152/japplphysiol.00927.2001

[37] Li H, Qiang Y, Wang L, Wang G, Yi J, et al (2013) Repair of lipopolysaccharide-induced acute lung injury in mice by endothelial progenitor cells, alone and in combination with simvastatin. Chest 144: 876–886. 23539119 10.1378/chest.12-2429

[38] Osorio F, Lambrecht B, Janssens S (2013) The UPR and lung disease. Semin Immunopathol 35: 293–306. 23536202 10.1007/s00281-013-0368-6

[39] Guzman E, Maher M, Temkin A, Pitts T, Wright A (2013) Spongiatriol inhibits nuclear factor kappa B activation and induces apoptosis in pancreatic cancer cells. Mar Drugs 11: 1140–1151. 23549285 10.3390/md11041140 PMC3705394

[40] Rentsendorj O, Mirzapoiazova T, Adyshev D, Servinsky LE, Renne T, et al (2008) Role of vasodilator-stimulated phosphoprotein in cGMP-mediated protection of human pulmonary artery endothelial barrier function. Am J Physiol Lung Cell Mol Physiol 294: L686–697. 18281604 10.1152/ajplung.00417.2007

[41] Lee S, Chung CY (2009) Role of VASP phosphorylation for the regulation of microglia chemotaxis via the regulation of focal adhesion formation/maturation. Mol Cell Neurosci 42: 382–390. 19733667 10.1016/j.mcn.2009.08.010 PMC3904500

[42] Chen BY, Honig B (2010) VASP: a volumetric analysis of surface properties yields insights into protein-ligand binding specificity. PLoS Comput Biol 6.. 10.1371/journal.pcbi.1000881 PMC2930297 20814581

[43] Rodgers LS, Beam MT, Anderson JM, Fanning AS (2013) Epithelial barrier assembly requires coordinated activity of multiple domains of the tight junction protein ZO-1. J Cell Sci 126: 1565–1575. 23418357 10.1242/jcs.113399 PMC3647435

[44] Heneghan AF, Pierre JF, Gosain A, Kudsk KA (2014) IL-25 improves luminal innate immunity and barrier function during parenteral nutrition. Ann Surg 259: 394–400. 23426341 10.1097/SLA.0b013e318284f510 PMC3661688

